# Association between pericapsular nerve group (PENG) block and postoperative inflammatory indices and analgesic outcomes in total hip arthroplasty: a randomized controlled trial

**DOI:** 10.1186/s12871-026-04084-4

**Published:** 2026-08-05

**Authors:** Anna Perek, Serkan Tulgar, Tomasz Reysner, Katrzyna Wieczorowska – Tobis, Paweł Pietraszek, Justyna Marszałek – Buko, Malgorzata Reysner

**Affiliations:** 1 Department of Anesthesiology and Intensive Care, Poznan University of Medical Sciences, ul. 28 Czerwca 1956 135/147, Poznań, 61-701 Poland; 2 Bahcesehir University Medicalpark Goztepe Hospital, Istanbul, Türkiye; 3 Department of Palliative Medicine, Poznan University of Medical Sciences, Poznań, Poland

**Keywords:** Pericapsular nerve group block, PENG block, Total hip arthroplasty, Regional anesthesia, Systemic inflammation, Neutrophil-to-lymphocyte ratio, Platelet-to-lymphocyte ratio, Randomized controlled trial, Postoperative pain, Opioid consumption

## Abstract

**Background:**

Total hip arthroplasty (THA) induces a pronounced postoperative inflammatory response that contributes to pain, increased opioid requirements, and delayed recovery, particularly in older adults. Although the pericapsular nerve group (PENG) block provides effective analgesia, its impact on systemic inflammation remains insufficiently defined.

**Methods:**

In this prospective, randomized, quadruple-blinded controlled trial, patients aged ≥ 60 years undergoing elective unilateral THA performed under spinal anesthesia were randomized (1:1) to receive an ultrasound-guided PENG block with ropivacaine or a sham block. The primary outcome was the neutrophil-to-lymphocyte ratio (NLR) at 12 h postoperatively. Secondary outcomes included platelet-to-lymphocyte ratio (PLR) at 12, 24, and 48 h, postoperative pain scores, time to first opioid administration, and cumulative opioid consumption within 48 h.

**Results:**

Sixty patients completed the study (30 per group). Baseline inflammatory markers were comparable between groups. At 12 h, NLR was significantly lower in the PENG group than in the control group (5.98 ± 1.42 vs. 9.60 ± 1.83; adjusted *p* < 0.0001), with sustained reductions at 24 h (2.47 ± 0.77 vs. 5.71 ± 1.74; adjusted *p* < 0.0001) and 48 h (1.84 ± 0.71 vs. 3.53 ± 1.08; adjusted *p* < 0.0001). PLR was also significantly lower in the PENG group at all postoperative time points (all adjusted *p* < 0.0001), with a significantly different postoperative trajectory between groups over time. The PENG block significantly prolonged time to first opioid administration (15.0 ± 1.5 vs. 9.3 ± 1.7 h; *p* < 0.001) and reduced cumulative 48-h opioid consumption (13.2 ± 2.7 vs. 20.8 ± 3.2 mg; *p* < 0.001). Pain scores at rest and during mobilization were consistently lower in the PENG group throughout the first 24 h.

**Conclusions:**

In older adults undergoing THA, the PENG block was associated with lower postoperative inflammatory indices, improved pain control, and reduced opioid requirements. Because NLR and PLR are indirect inflammatory markers, these findings should be interpreted cautiously and viewed as associative rather than mechanistic. Further studies are required to determine whether these physiological differences translate into improved clinical recovery outcomes.

**Trial registration:**

ClinicalTrials.gov (NCT07023107), registered on 8 June 2025.

## Introduction

Total hip arthroplasty (THA) is a highly effective procedure for restoring mobility and quality of life, but it is accompanied by a pronounced surgical stress response, particularly in older adults [1]. Tissue injury, neuroendocrine activation, and perioperative pain collectively trigger a systemic inflammatory cascade that may contribute to increased postoperative pain, delayed mobilization, higher opioid requirements, and adverse recovery trajectories [2]. Attenuating this inflammatory response has therefore become an essential goal of modern perioperative care, especially within enhanced recovery protocols [3, 4].

Regional anesthesia is a cornerstone of multimodal analgesia in THA and provides benefits beyond pain control [5]. By reducing afferent nociceptive input and sympathetic activation, regional techniques may also modulate postoperative immune and inflammatory responses [6]. Among readily available biomarkers of systemic inflammation, the neutrophil-to-lymphocyte ratio (NLR) and platelet-to-lymphocyte ratio (PLR) have gained increasing attention [7–10]. However, these indices are indirect and nonspecific markers that reflect systemic stress and immune balance rather than direct inflammatory pathways. Also, it remains unclear whether the associations between regional anesthesia and postoperative inflammatory responses are uniform across different surgical procedures or depend on the anatomical origin of nociceptive input. Hip and knee arthroplasty differ substantially in terms of tissue injury, innervation patterns, and postoperative inflammatory response, which may influence the extent of systemic immune modulation. Understanding whether regional techniques exert procedure-specific effects is essential for optimizing multimodal perioperative strategies [6, 11–13]. These indices, derived from routine blood counts, are sensitive indicators of surgical stress and have been associated with pain intensity, postoperative complications, and delayed functional recovery after major orthopedic procedures.

The pericapsular nerve group (PENG) block is an established regional anesthesia technique designed to target the sensory innervation of the anterior hip capsule, including articular branches of the femoral, obturator, and accessory obturator nerves [14]. Its anatomical specificity allows effective analgesia while largely preserving quadriceps motor function, which is critical for early mobilization after THA. Clinical studies have demonstrated that the PENG block provides effective postoperative analgesia and reduces opioid consumption when used as part of a multimodal strategy [15]. However, despite its widespread adoption in hip surgery, the broader physiological effects of the PENG block—particularly its influence on the postoperative systemic inflammatory response—remain insufficiently characterized.

In particular, the PENG block offers a unique model for investigating the relationship between targeted nociceptive pathways and systemic inflammatory responses, as it selectively anesthetizes the anterior hip capsule, a key contributor to post-hip arthroplasty pain [11, 12, 16]. Whether this anatomical specificity translates into measurable differences in systemic inflammation has not been clearly established [14, 17].

Older patients undergoing THA represent a population at increased risk of exaggerated inflammatory responses and opioid-related adverse effects [18]. In this context, regional techniques associated with improved analgesia and lower postoperative inflammatory indices may offer particular benefit [2]. While previous investigations have explored the impact of neuraxial anesthesia and certain peripheral nerve blocks on inflammatory markers [11], high-quality prospective data specifically evaluating the effect of the PENG block on postoperative inflammatory indices are lacking. Moreover, most available studies have focused primarily on analgesic endpoints, without integrating objective biomarkers of immune response [14, 15, 19].

The present randomized, quadruple-blinded controlled trial was therefore designed to evaluate the effect of the PENG block on early postoperative systemic inflammation in older adults undergoing total hip arthroplasty under spinal anesthesia. The primary objective was to assess differences in the neutrophil-to-lymphocyte ratio 12 h after surgery. Secondary objectives included analysis of the temporal course of NLR and PLR, postoperative pain intensity, opioid consumption, and time to first opioid administration. We hypothesized that, in addition to improving analgesic outcomes, the PENG block would be associated with lower postoperative inflammatory indices compared with a sham procedure.

## Methods

### Study design and ethical approval

This study was a prospective, randomized, parallel-group, quadruple-blinded, controlled clinical trial evaluating the effect of the pericapsular nerve group (PENG) block on postoperative systemic inflammation and analgesic outcomes in older adults undergoing total hip arthroplasty (THA). The trial was conducted at the Poznan University of Medical Sciences (Poznań, Poland). Ethical approval was granted by the Bioethics Committee at the Poznan University of Medical Sciences on 12 March 2025 (Approval No. 107/24). The study was prospectively registered at ClinicalTrials.gov on 8 June 2025 under the identifier NCT07023107, before the enrollment of the first participant.

Patient recruitment began on 16 June 2025 and continued until 3 December 2025, when the planned sample size was reached. All follow-up assessments were completed by 9 December 2025, which served as both the primary completion date and the final study completion date. All participants provided written informed consent before enrollment. The study was conducted in accordance with the Declaration of Helsinki and Good Clinical Practice (GCP) standards. This study was conducted and reported in accordance with the CONSORT 2010 guidelines for randomized controlled trials.

### Participants

Participants were eligible for inclusion if they were 60 to 100 years of age, were scheduled for elective unilateral total hip arthroplasty performed under spinal anesthesia, and had an ASA physical status of I–III. Additional inclusion criteria required that each patient be able to provide informed consent and reliably report pain scores. Patients were excluded if they had a history of bleeding diathesis or were receiving therapeutic anticoagulation. Individuals with chronic pain unrelated to osteoarthritis (e.g., non-arthritic chronic pain conditions or long-term opioid use) were excluded, whereas osteoarthritis-related pain was expected and allowed. Further exclusion criteria included infection at the planned block site, use of general anesthesia as the primary anesthetic, and refusal to participate in the study.

### Randomization and blinding

Participants were randomized 1:1 to receive either the active PENG block or a sham block. Randomization was performed using a computer-generated sequence created in nQuery Advisor (Statistical Solutions, Boston, MA, USA)**,** employing permuted block randomization to maintain balanced group sizes throughout enrollment. The allocation sequence was prepared by a statistician independent of all clinical activities and stored in sequentially numbered, sealed, opaque envelopes, thereby ensuring complete allocation concealment until the block procedure. These were opened only by the anesthesiologist performing the block immediately before the procedure.

Participant-, care provider-, assessor-, and analyst-blinding were maintained throughout the study. Participants were blinded because both interventions used identical positioning, ultrasound guidance, needle insertion, and communication. Ropivacaine and saline were prepared in identical syringes by an independent research nurse.

All perioperative care providers—including surgeons, anesthesiologists not involved in the block, nurses, and ward staff—remained unaware of group allocation and followed standardized postoperative protocols. Investigators responsible for data collection and clinical monitoring were also blinded and performed all assessments using coded participant identifiers.

Outcome assessors and laboratory personnel analyzed inflammatory markers and clinical endpoints under fully anonymized conditions. Statistical analyses were performed using coded group allocation identifiers, and the analyst remained blinded until completion of the predefined statistical analysis plan. The anesthesiologist performing the block was the only unblinded individual and had no role in postoperative care, data collection, or study assessments.

Blinding was preserved until all participants completed follow-up and the final dataset was locked. Unmasking occurred only after completion of the predefined statistical analysis plan.

### Interventions

#### Spinal anesthesia (standardized for all participants)

All patients received spinal anesthesia before the study intervention to ensure a uniform baseline anesthetic technique. Spinal anesthesia was performed at the L2–L3 or L3–L4 interspace using a 25G or 27G Whitacre needle, and 0.5% ropivacaine (12–15 mg) was administered intrathecally according to departmental standards. Perioperative medications, fluid therapy, and surgical technique were standardized across groups to minimize variability. No intraoperative systemic opioids were routinely administered after the establishment of adequate spinal anesthesia. Light intravenous sedation with propofol or midazolam was permitted when clinically indicated and followed standardized institutional practice.

#### PENG block group

Patients randomized to the intervention group received an ultrasound-guided PENG block immediately following spinal anesthesia. A low-frequency curvilinear transducer was used to identify the anterior inferior iliac spine, iliopubic eminence, and psoas tendon. An in-plane lateral-to-medial needle approach was used, and after confirming the correct plane, 20 mL of 0.2% ropivacaine was injected incrementally. The technique followed established protocols to maintain sensory targeting and reduce unintended femoral motor involvement.

#### Control group

Participants assigned to the control group underwent a sham procedure that replicated all elements of the active block except the anesthetic agent. The same ultrasound transducer, needle type, patient positioning, and procedural timing were used. The needle was advanced to the same sonographic landmarks, and 20 mL of 0.9% sodium chloride was injected to mimic the sensory experience of an active block. Duration, communication, and procedural steps were standardized to preserve blinding.

### Postoperative analgesia protocol

All patients received an identical multimodal postoperative analgesia regimen. Acetaminophen 1 g IV every 6 h and metamizole 1 g IV every 6 h were administered as scheduled medications, with ibuprofen 400 mg IV every 8 h unless contraindicated. Rescue opioid therapy consisted of 5 mg IV oxycodone administered when the pain score exceeded NRS ≥ 4. Antiemetic prophylaxis with 8 mg IV ondansetron was provided as needed. Early mobilization was encouraged in accordance with standardized physiotherapy protocols, beginning approximately 10 h after surgery. No perioperative corticosteroids were administered. Perioperative corticosteroids were intentionally avoided to minimize potential confounding effects on postoperative inflammatory biomarker assessment.

### Outcomes

The *primary outcome* of the study was the *neutrophil-to-lymphocyte ratio (NLR)* measured *12 h after surgery*. NLR was obtained from a peripheral venous blood sample collected at the specified time point. The ratio was calculated as the absolute neutrophil count divided by the absolute lymphocyte count, as determined by automated hematology analysis. NLR was selected as the primary endpoint because it is an established, sensitive biomarker of surgical stress and systemic inflammation, and its early postoperative trajectory correlates with the extent of tissue injury and physiological stress responses.

### Secondary outcomes

Secondary inflammatory outcomes included the *platelet-to-lymphocyte ratio (PLR)* measured at *12, 24, and 48 h* after surgery, as well as *NLR values at 24 and 48 h*, enabling evaluation of the temporal profile of postoperative systemic inflammation.

Analgesic outcomes included the *time to first opioid administration*, defined as the number of hours elapsed between completion of surgery and the first dose of rescue opioid, and the *cumulative opioid consumption* during the first 48 h postoperatively, calculated in standardized oral morphine equivalents. Cumulative opioid consumption during the first 48 h postoperatively was converted to standardized oral morphine equivalents (OME). For patients receiving intravenous oxycodone, conversion was performed using an equianalgesic ratio of 1 mg IV oxycodone = 3 mg oral morphine. This conversion ratio was selected based on institutional practice and published equianalgesic conversion tables. We acknowledge that alternative conversion factors have been reported in the literature; however, because all opioid consumption comparisons were performed using the same conversion factor across groups, the relative between-group differences remain unchanged.

Postoperative *pain intensity* was assessed using an 11-point Numerical Rating Scale (NRS; 0 = no pain, 10 = worst pain imaginable) at 4, 8, 12, and 24 h after surgery, both at rest and during mobilization, to capture dynamic and functional pain profiles during the early postoperative period.

All clinical assessments, including pain scoring, opioid administration logs, and timing measurements, were performed by outcome assessors who remained blinded to group allocation throughout follow-up.

### Sample size calculation

The sample size was determined a priori based on the prespecified primary endpoint, namely the between-group difference in neutrophil-to-lymphocyte ratio (NLR) at 12 h after surgery. Estimates of the expected effect size and variability were informed by a pilot dataset obtained in a small group of patients (*n* = 5 per group) treated at our institution prior to study initiation. In this preliminary sample, the mean NLR at 12 h was approximately 6.8 ± 1.7 in the control group and 5.3 ± 1.6 in the PENG group, corresponding to an absolute between-group difference of approximately 1.5 units. Based on these observations, and assuming a common standard deviation of 1.8, a two-sided alpha level of 0.05 and 90% power, 29 participants per group were required to detect a statistically significant difference between groups. To account for potential attrition and to preserve balanced allocation, the final sample size was increased to 30 participants per group (total *n* = 60). Given the limited size of the pilot dataset and the absence of a well-established minimal clinically important difference for NLR in this setting, the selected effect size should be interpreted as exploratory and hypothesis-generating.

### Data collection and laboratory methods

Peripheral blood samples were obtained at baseline and at 12, 24, and 48 h postoperatively. Complete blood counts with differentials were analyzed using certified automated hematology analyzers. NLR and PLR were calculated immediately after laboratory processing. Pain scores, opioid administration times, and cumulative opioid use were collected from electronic medical records by blinded assessors.

### Statistical analysis

Statistical analyses were performed using GraphPad Prism version 11.0.0 (GraphPad Software, San Diego, CA, USA). All analyses were conducted according to the intention-to-treat principle.

Continuous variables were assessed using visual inspection of histograms and Q–Q plots and are presented as mean ± standard deviation (SD). Normality was additionally assessed using the Shapiro–Wilk test. Baseline characteristics were compared using the independent-samples t-test or Fisher’s exact test, as appropriate.

The prespecified primary endpoint was the between-group difference in neutrophil-to-lymphocyte ratio (NLR) at 12 h after surgery, analyzed using an independent-samples t-test with reporting of mean difference and 95% confidence interval (CI).

For repeated measurements of inflammatory markers (NLR and PLR), linear mixed-effects models (restricted maximum likelihood, REML) were used, with time, treatment group, and their interaction included as fixed effects, and subject as a random effect to account for within-subject correlations. A compound symmetry covariance structure was selected a priori because repeated measurements were obtained at fixed postoperative intervals and correlations between repeated observations were expected to remain relatively stable over time. Pain scores (NRS) were analyzed using between-group comparisons at individual postoperative time points. When significant overall effects were detected, post hoc pairwise comparisons between groups at individual time points were performed using Šídák correction for multiple testing. Two-sided *p*-values < 0.05 were considered statistically significant. As the study was powered for the primary endpoint at 12 h, repeated-measures and secondary outcome analyses should be interpreted as supportive and exploratory, particularly given the potential risk of type I error inflation associated with multiple comparisons.

## Results

**Fig. 1:**
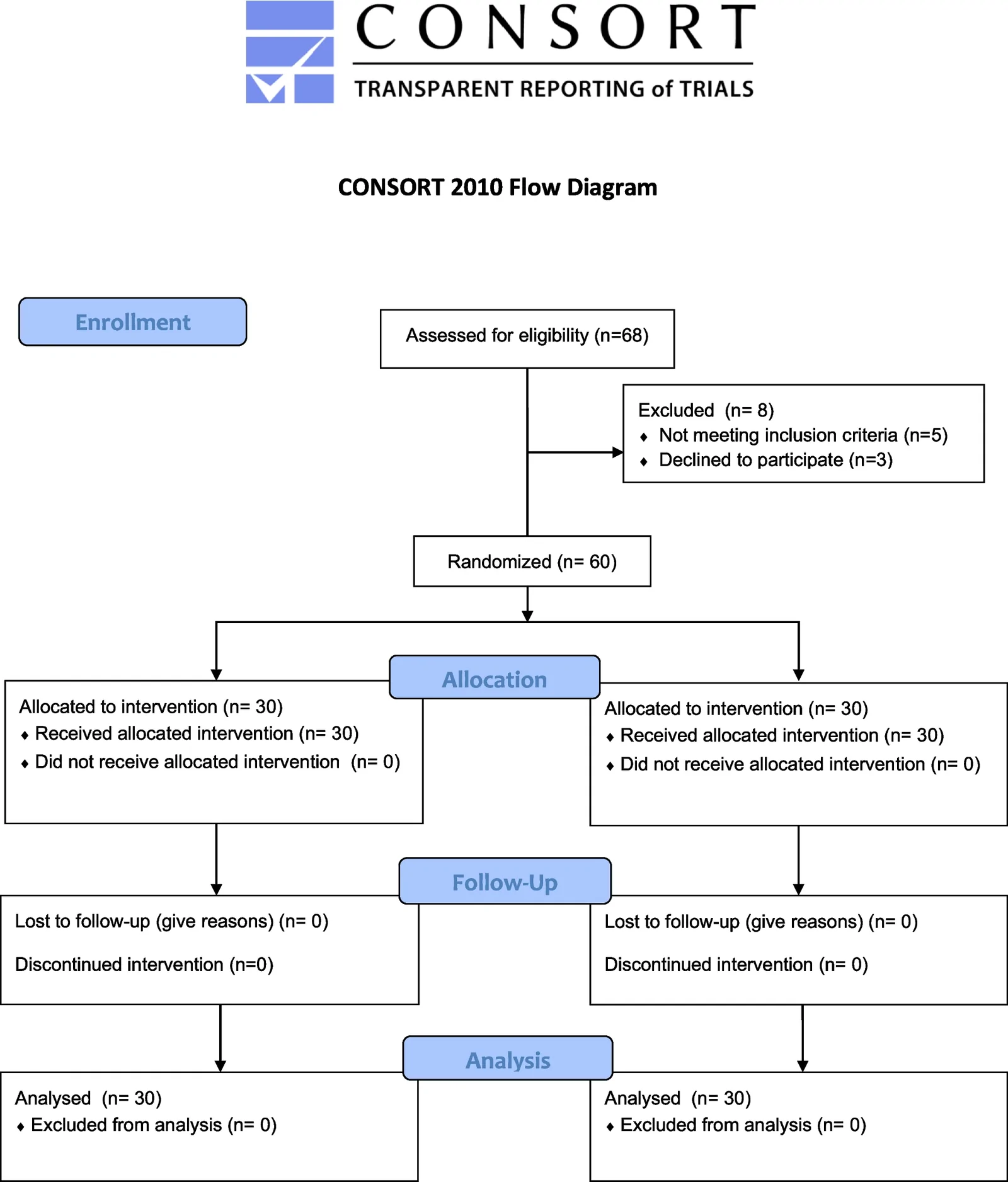
CONSORT flow diagram for participant enrollment, randomization, intervention allocation, follow-up, and analysis

A total of 68 patients were screened; 60 met eligibility criteria and provided consent to participate. Eight patients were excluded before randomization: five did not meet the inclusion criteria, and three declined to participate. Sixty patients were randomly assigned to the Control group (*n* = 30) and the PENG group (*n* = 30). All participants received the assigned intervention, completed follow-up, and were included in the final analysis. No protocol deviations or losses to follow-up occurred (Fig. 1).

Baseline demographic and perioperative characteristics were similar between groups, including age, sex, ASA physical status, BMI, surgical duration, and spinal anesthesia level (Table 1).

**Table 1:** Baseline characteristics of the study population

Characteristic	Control (*n* = 30)	PENG (*n* = 30)
Age, years (mean ± SD)	72.3 ± 5.1	71.8 ± 4.8
Sex, male/female	15/15	14/16
BMI, kg/m^2^ (mean ± SD)	30.2 ± 3.8	29.7 ± 3.5
ASA physical status II — no. (%)	17 (57%)	18 (60%)
ASA physical status III — no. (%)	13 (43%)	12 (40%)
Duration of surgery, min (mean ± SD)	62 ± 9	60 ± 10
Side of surgery (right/left)	16/14	15/15
Implant type (cemented/uncemented)	12/18	11/19
Spinal anesthesia level (L2–3/L3–4)	13/17	14/16
Baseline pain at rest (NRS 0–10)	2.1 ± 0.7	2.0 ± 0.6
Baseline pain during mobilization (NRS 0–10)	3.0 ± 0.9	2.9 ± 0.8

### Primary outcome: neutrophil-to-lymphocyte ratio (NLR)

Baseline inflammatory markers were similar between groups, with no significant differences in preoperative NLR (0.94 ± 0.39 vs 0.99 ± 0.42; *p* = 0.9684), confirming initial group consistency. Across postoperative follow-up, NLR values remained consistently lower in the PENG group compared with the control group.

At the prespecified 12-h primary endpoint, mean NLR was significantly lower in the PENG group (5.98 ± 1.42) compared with the control group (9.60 ± 1.83), corresponding to a mean difference of − 3.61 (95% CI, − 4.70 to − 2.53; adjusted *p* < 0.0001).

**Fig. 2:**
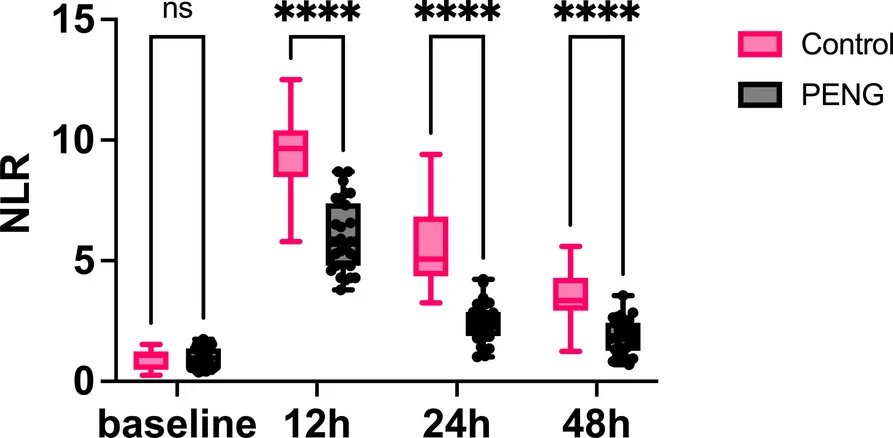
Neutrophil-to-lymphocyte ratio (NLR) at 12, 24, and 48 h after surgery in the control and PENG groups

This difference remained significant at 24 h (2.47 ± 0.77 vs. 5.71 ± 1.74; mean difference − 3.24, 95% CI − 4.15 to − 2.33; adjusted *p* < 0.0001) and at 48 h (1.84 ± 0.71 vs. 3.53 ± 1.08; mean difference − 1.69, 95% CI − 2.30 to − 1.09; adjusted *p* < 0.0001), as shown in Fig. 2 and Table 2.

**Table 2:** Inflammatory markers

Outcome	Control group	PENG group	Mean difference (95%Cl)	Adjusted *p*-value
NLR baseline	0.94 ± 0.39	0.99 ± 0.42	0.06 (− 0.21 to 0.33)	0.9684
NLR 12 h	9.60 ± 1.83	5.98 ± 1.42	− 3.61 (− 4.70 to − 2.53)	< 0.0001
NLR 24 h	5.71 ± 1.74	2.47 ± 0.77	− 3.24 (− 4.15 to − 2.33)	< 0.0001
NLR 48 h	3.53 ± 1.08	1.84 ± 0.71	− 1.69 (− 2.30 to − 1.09)	< 0.0001
PLR baseline	173.3 ± 36.5	178.6 ± 50.2	5.31 (− 29.44 to 40.06)	0.9921
PLR 12 h	273.4 ± 51.3	203.3 ± 45.6	− 70.14 (− 104.9 to − 35.4)	< 0.0001
PLR 24 h	274.9 ± 75.1	202.2 ± 56.9	− 72.72 (− 107.5 to − 37.97)	< 0.0001
PLR 48 h	215.7 ± 64.9	150.0 ± 35.6	− 65.73 (− 100.5 to − 31.0)	< 0.0001

*PENG* Pericapsular Nerve Group block, *NLR* Neutrophil-to-Lymphocyte Ratio, a biomarker of systemic inflammation and surgical stress, *PLR* Platelet-to-Lymphocyte Ratio, an additional inflammatory marker, *CI* Confidence Interval

Data are presented as mean ± SD. Adjusted *p*-values were obtained using Šídák-corrected pairwise comparisons. CI, confidence interval

### Secondary outcomes

#### PLR

Preoperative PLR values were comparable between groups (173.3 ± 36.5 vs 178.6 ± 50.2; adjusted p = 0.9921). Linear mixed-effects analysis demonstrated significant effects of time (*p* < 0.0001), treatment group (*p* < 0.0001), and a significant time-by-group interaction (*p* = 0.0003), indicating that the trajectory of postoperative PLR differed between groups.

At 12 h, PLR was significantly lower in the PENG group (203.3 ± 45.6) compared with the control group (273.4 ± 51.3), corresponding to a mean difference of − 70.14 (95% CI, − 104.9 to − 35.4; adjusted *p* < 0.0001).

**Fig. 3:**
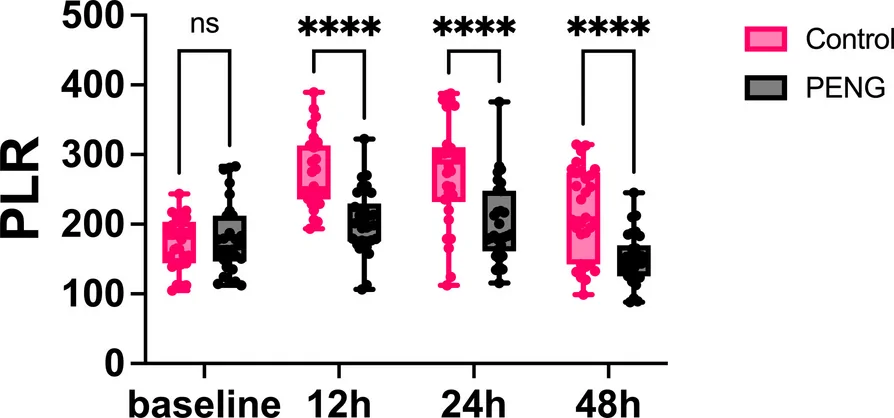
Platelet-to-lymphocyte ratio (PLR) at 12, 24, and 48 h after surgery in the control and PENG groups

This difference remained significant at 24 h (202.2 ± 56.9 vs. 274.9 ± 75.1; mean difference − 72.72, 95% CI − 107.5 to − 37.97; adjusted *p* < 0.0001) and at 48 h (150.0 ± 35.6 vs. 215.7 ± 64.9; mean difference − 65.73, 95% CI − 100.5 to − 31.0; adjusted *p* < 0.0001), as illustrated in Fig. 3 and Table 2.

#### Opioid-related outcomes

The PENG block resulted in a significant delay in the need for postoperative opioid analgesia. The mean time to first opioid administration was 15.0 ± 1.5 h in the PENG group versus 9.3 ± 1.7 h in the Control group (*p* < 0.001), corresponding to a prolongation of 5.7 h (95% CI, 4.7–6.7). Cumulative opioid use over 48 h was also significantly lower in the PENG group, with consumption of 13.2 ± 2.7 mg compared with 20.8 ± 3.2 mg in Controls (*p* < 0.001). The between-group difference was − 7.6 mg (95% CI, − 9.0 to − 6.2), indicating reduced opioid consumption, as seen in Table 3.

**Table 3:** Secondary outcomes

Outcome	Control group	PENG group	*p*-value	Mean difference (95% CI)
OPIOID Consumption				
Time to first rescue opioid (h)	9.3 ± 1.7	15.0 ± 1.5	< 0.001	5.7 [4.7 to 6.7]
Total 48 h opioid consumption (mg OME)	20.8 ± 3.2	13.2 ± 2.7	< 0.001	−7.6 [−9.0 to −6.2]
PAIN SCORES				
mobilization				
Pain score at 4 h	5.0 ± 0.8	3.3 ± 0.7	< 0.001	−1.7 [−2.2 to −1.2]
Pain score at 8 h	4.7 ± 0.9	2.9 ± 0.6	< 0.001	−1.8 [−2.3 to −1.3]
Pain score at 12 h	4.5 ± 1.0	2.7 ± 0.8	< 0.001	−1.8 [−2.4 to −1.2]
Pain score at 24 h	3.9 ± 0.8	2.2 ± 0.7	< 0.001	−1.7 [−2.2 to −1.2]
at rest				
Pain score at 4 h	3.5 ± 0.9	2.5 ± 0.8	< 0.001	−1.0 [−1.4 to −0.6]
Pain score at 8 h	3.2 ± 0.8	2.3 ± 0.7	< 0.001	−0.9 [−1.3 to −0.5]
Pain score at 12 h	2.8 ± 0.7	2.0 ± 0.6	< 0.001	−0.8 [−1.1 to −0.5]
Pain score at 24 h	2.0 ± 0.6	1.5 ± 0.5	< 0.001	−0.5 [−0.7 to −0.3]

*PENG* Pericapsular Nerve Group block, *THA* Total Hip Arthroplasty, *NLR* Neutrophil-to-Lymphocyte Ratio, a biomarker of systemic inflammation and surgical stress, *PLR* Platelet-to-Lymphocyte Ratio, an additional inflammatory marker, *CI* Confidence Interval. *h* Hours, *mg* Milligrams

All values are presented as mean ± standard deviation (SD)

*p*-values represent the statistical significance of differences between the Control and the PENG groups

Mean difference (95% CI) indicates the absolute difference in means between groups with the corresponding 95% confidence interval

#### Pain outcomes

Pain during mobilization was lower in the PENG group than in the control group at all assessed postoperative time points. At 4, 8, 12, and 24 h, mean pain scores during mobilization were 3.3 ± 0.7, 2.9 ± 0.6, 2.7 ± 0.8, and 2.2 ± 0.7 in the PENG group, compared with 5.0 ± 0.8, 4.7 ± 0.9, 4.5 ± 1.0, and 3.9 ± 0.8 in the control group, respectively (all *p* < 0.001).

Pain at rest was also lower in the PENG group at all assessed time points. At 4, 8, 12, and 24 h, mean resting pain scores were 2.5 ± 0.8, 2.3 ± 0.7, 2.0 ± 0.6, and 1.5 ± 0.5 in the PENG group, compared with 3.5 ± 0.9, 3.2 ± 0.8, 2.8 ± 0.7, and 2.0 ± 0.6 in the control group, respectively (all *p* < 0.001).

To summarize the postoperative pain burden, mean NRS values across all time points were calculated. During mobilization, the overall mean NRS was lower in the PENG group compared with the control group (2.78 vs. 4.53), corresponding to an absolute difference of 1.75 points. At rest, the overall mean NRS was also lower in the PENG group (2.08 vs. 2.88), with a difference of 0.80 points.

No adverse events or block-related complications were observed, although the study was not powered to detect rare complications.

## Discussion

In this randomized, quadruple-blinded controlled trial, the pericapsular nerve group (PENG) block was associated with lower early postoperative inflammatory indices and improved analgesic outcomes in older adults undergoing total hip arthroplasty (THA). The study suggests that the PENG block may be associated not only with modulation of nociceptive pathways but also with differences in the broader biological stress response to surgery [20]. To our knowledge, this is among the first prospective trials to show a sustained reduction in postoperative inflammatory biomarkers—specifically NLR and PLR—after a single-shot regional anesthesia technique.

These findings should be interpreted as associative and hypothesis-generating rather than mechanistic. The proposed pathways linking nociceptive input and immune activation remain speculative and were not directly assessed in this study.

Importantly, our findings suggest that the magnitude of systemic inflammatory modulation may depend on the anatomical origin of nociceptive input [6, 21, 22]. The PENG block selectively targets the anterior hip capsule, which plays a central role in nociceptive signaling during total hip arthroplasty [11, 21, 23]. This may contribute to differences in nociceptive processing and postoperative stress responses, thereby influencing systemic inflammatory markers such as NLR and PLR [6, 24, 25].

The primary endpoint, NLR at 12 h, was markedly lower in the PENG group, suggesting an association with lower early postoperative inflammatory indices [26]. This benefit persisted at 24 and 48 h, suggesting sustained differences in postoperative inflammatory marker trajectories [24]. Importantly, baseline inflammatory markers were well balanced between groups, supporting that the observed postoperative differences reflect the effect of the intervention rather than pre-existing variability. Since elevated postoperative NLR is associated with increased tissue injury, worse pain, and delayed recovery, the decreases observed in this trial indicate a meaningful physiologic advantage [16, 23]. The PLR findings reinforce this effect, with consistently lower values across all time points. The magnitude of between-group differences and the non-overlapping confidence intervals indicate a consistent association with lower inflammatory indices. However, the present study was not designed to evaluate underlying biological mechanisms, and any explanation linking these findings to nociceptive, sympathetic, or opioid-related pathways remains speculative [25]. The anatomical specificity of the PENG block, which targets sensory innervation of the anterior hip capsule while sparing motor fibers, may contribute to these consistent results [14]. However, no validated minimal clinically important difference has been established for postoperative NLR or PLR values in patients undergoing THA. Therefore, the observed between-group differences should primarily be interpreted as indicators of differing physiological stress responses rather than definitive evidence of improved clinical recovery.

Analgesic outcomes further support the physiological benefits of the PENG block. Time to first opioid administration was prolonged by nearly six hours, and cumulative opioid consumption was markedly reduced [15]. For older adults—who are more susceptible to opioid-related adverse effects—these reductions are clinically significant [21]. Pain scores during mobilization showed substantial and consistent improvement, with reductions of nearly two NRS points across the first 24 h. Because dynamic pain is a major determinant of early mobility after THA, these improvements may be clinically relevant, although functional outcomes were not directly assessed in this study [22, 27]. Pain at rest was also lower in the PENG group, although the absolute difference was minor. Together, the observed analgesic effects and lower postoperative inflammatory indices may be interrelated, given the well-established relationship among pain, sympathetic activation, and immune signaling [12, 17].

These observations support the concept that associations between regional anesthesia and postoperative inflammatory responses are not uniform and may vary with surgical context, tissue injury characteristics, and the specific neural pathways involved. This highlights the importance of considering procedure-specific mechanisms when selecting regional techniques as part of multimodal perioperative care [6, 14, 27].

The mechanisms linking regional anesthesia and inflammation are complex and not fully understood. By reducing nociceptive input from the hip capsule—one of the most densely innervated articular structures—the PENG block may dampen neuroinflammatory feedback loops that amplify postoperative immune activation [13]. Additionally, the opioid-sparing effect likely contributes to reduced immune dysregulation, as opioids can influence leukocyte function and inflammatory mediator release [28, 29].

This concept aligns with emerging evidence suggesting that the interaction between nociceptive pathways and immune activation is a key determinant of postoperative inflammatory response [25, 27]. Future studies incorporating cytokine profiling (e.g., IL-6, CRP) are required to determine whether these associations reflect true biological modulation [30].

This study has limitations. It was conducted at a single center, which may limit generalizability, although uniform surgical and anesthetic protocols enhance internal validity. NLR and PLR, while valuable biomarkers, do not capture specific cytokine pathways; future studies incorporating markers such as IL-6 or CRP would provide deeper mechanistic insight. The 48-h follow-up does not allow assessment of long-term functional or clinical outcomes. Finally, although a sham block was used, saline injection may have produced minimal mechanical effects. The study did not include objective functional recovery measures such as the Timed Up and Go test, 5-m walk test, or quadriceps strength assessment, which could provide additional insight into the motor-sparing properties of the PENG block. Furthermore, validated recovery metrics such as the Quality of Recovery-15 (QoR-15) questionnaire were not assessed, limiting the ability to correlate physiological and patient-centered outcomes. Also, saline injection may have exerted minor mechanical or pressure-related effects on nociception; therefore, the observed differences may represent a conservative estimate of the true effect. Functional recovery and mobilization were not directly assessed and therefore cannot be inferred from the present data. Effect sizes, such as Cohen’s d, were not reported because the study was primarily designed to estimate between-group mean differences and to provide confidence intervals for clinically interpretable outcomes. In addition, the sample size calculation was based on a small pilot dataset (*n* = 5 per group), which may have introduced uncertainty into the effect size estimate used for study planning.

## Conclusion

The PENG block was associated with lower postoperative inflammatory indices, improved pain control, and reduced opioid requirements in older patients undergoing THA. These findings should be interpreted cautiously, as the inflammatory markers used are indirect and mechanistic pathways were not directly assessed. Further studies are required to confirm these associations and to explore their clinical relevance.

## Data Availability

The datasets generated and/or analyzed during the current study are available from the corresponding author on reasonable request.

## Abbreviations

PENG: Pericapsular Nerve Group
THA: Total Hip Arthroplasty
NLR: Neutrophil-to-Lymphocyte Ratio
PLR: Platelet-to-Lymphocyte Ratio
OME: Oral Morphine Equivalent
CI: Confidence Interval
NRS: Numerical Rating Scale

## References

[1] Karabulut N, Gürçayır D, Abi Ö, KızıloğluAğgül B, Söylemez N. Does surgery cause anxiety, stress and fear in geriatric patients? Psychogeriatrics. 2023;23(5):808–14. 37433670 10.1111/psyg.13000

[2] Reysner T, Wieczorowska-Tobis K, Kowalski G, Grochowicka M, Pyszczorska M, Mularski A, et al. The influence of regional anesthesia on the systemic stress response. Reports. 2024;7(4):89. 40757696 10.3390/reports7040089 PMC12199975

[3] Xing N, Wang H, Huang Y, Peng J. Enhanced recovery after surgery program alleviates neutrophil-to-lymphocyte ratio and platelet-to-lymphocyte ratio in patients undergoing gynecological surgery. Front Med. 2023;10:1057923. 10.3389/fmed.2023.1057923 PMC10150635 37138751

[4] Carli F. Physiologic considerations of Enhanced Recovery After Surgery (ERAS) programs: implications of the stress response. Can J Anesth. 2015;62(2):110–9. 10.1007/s12630-014-0264-0. 25501695

[5] Mancel L, Van Loon K, Lopez AM. Role of regional anesthesia in Enhanced Recovery After Surgery (ERAS) protocols. Curr Opin Anesthesiol. 2021;34(5):616–25. 10.1097/ACO.0000000000001048 34325463

[6] Cusack B, Buggy D. Anaesthesia, analgesia, and the surgical stress response. BJA Educ. 2020;20(9):321–8. 33456967 10.1016/j.bjae.2020.04.006 PMC7807970

[7] Ghobadi H, Mohammadshahi J, Javaheri N, Fouladi N, Mirzazadeh Y, Aslani MR. Role of leukocytes and systemic inflammation indexes (NLR, PLR, MLP, dNLR, NLPR, AISI, SIR-I, and SII) on admission predicts in-hospital mortality in non-elderly and elderly COVID-19 patients. Front Med. 2022;9:916453. 10.3389/fmed.2022.916453 PMC9434555 36059829

[8] Domagalska M, Ciftsi B, Janusz P, Reysner T, Kolasinski J, Wieczorowska-Tobis K, et al. The neutrophil-to-lymphocyte ratio (NLR) and platelet-to-lymphocyte ratio (PLR) levels following erector spinae plane block (ESPB) in posterior lumbar decompression: a randomized, controlled trial. Eur Spine J. 2023;32(12):4192–9. 37668689 10.1007/s00586-023-07913-z

[9] Domagalska M, Reysner T, Kowalski G, Daroszewski P, Mularski A, Wieczorowska-Tobis K. Pain management, functional recovery, and stress response expressed by NLR and PLR after the iPACK block combined with adductor canal block for total knee arthroplasty—a prospective, randomised, double-blinded clinical trial. J Clin Med. 2023;12(22):7088. 38002702 10.3390/jcm12227088 PMC10672046

[10] Kriplani A, Pandit S, Chawla A, de la Rosette JJ, Laguna P, Jayadeva Reddy S, et al. Neutrophil–lymphocyte ratio (NLR), platelet–lymphocyte ratio (PLR) and lymphocyte–monocyte ratio (LMR) in predicting systemic inflammatory response syndrome (SIRS) and sepsis after percutaneous nephrolithotomy (PNL). Urolithiasis. 2022;50(3):341–8. 35246692 10.1007/s00240-022-01319-0 PMC9110452

[11] Jaruga K, Puścion-Jakubik A, Jakubów P. Regional anesthesia: a narrative review of impact on oxidative stress biomarkers. J Clin Med. 2025;14(21):7503. 41226899 10.3390/jcm14217503 PMC12608378

[12] Ji J, Huh Y, Ji RR. Inflammatory Mediators, Nociceptors, and Their Interactions in Pain. W: Neuroimmune interactions in pain: mechanisms and therapeutics. Springer; 2023. s. 87–119.

[13] Verdonk F, Einhaus J, Tsai AS, Hedou J, Choisy B, Gaudilliere D, et al. Measuring the human immune response to surgery: multiomics for the prediction of postoperative outcomes. Curr Opin Crit Care. 2021;27(6):717–25. 34545029 10.1097/MCC.0000000000000883 PMC8585713

[14] Domagalska M, Ciftci B, Reysner T, Kolasiński J, Wieczorowska-Tobis K, Kowalski G. Pain management and functional recovery after pericapsular nerve group (PENG) block for total hip arthroplasty: a prospective, randomized, double-blinded clinical trial. J Clin Med. 2023;12(15):4931. 37568331 10.3390/jcm12154931 PMC10420102

[15] Reysner T, Kowalski G, Reysner M, Mularski A, Daroszewski P, Wieczorowska-Tobis K. Functional recovery and pain control following pericapsular nerve group (PENG) block following hip surgeries: a systematic review and meta-analysis of randomised controlled trials. Arch Orthop Trauma Surg. 2025;145(1):1–15. 10.1007/s00402-025-05825-9 40105988

[16] Basu P, Maier C, Averitt DL, Basu A. NLR family pyrin domain containing 3 (NLRP3) inflammasomes and peripheral neuropathic pain-emphasis on microRNAs (miRNAs) as important regulators. Eur J Pharmacol. 2023;955:175901. 37451423 10.1016/j.ejphar.2023.175901

[17] Doménech-García V, Peirotén AR, Imaz ML, Palsson TS, Herrero P, Bellosta-López P. Not just sensitization: sympathetic mechanisms contribute to expand experimental referred pain. Korean J Pain. 2022;35(3):240–9. 35768979 10.3344/kjp.2022.35.3.240 PMC9251400

[18] Shellito AD, Dworsky JQ, Kirkland PJ, Rosenthal RA, Sarkisian CA, Ko CY, et al. Perioperative pain management issues unique to older adults undergoing surgery: a narrative review. Ann Surg Open. 2021;2(3):e072. 34870279 10.1097/AS9.0000000000000072 PMC8635081

[19] Amato PE, Coleman JR, Dobrzanski TP, Elmer DA, Gwathmey FW, Slee AE, et al. Pericapsular nerve group (PENG) block for hip arthroscopy: a randomized, double-blinded, placebo-controlled trial. Reg Anesth Pain Med. 2022;47(12):728–32. 10.1136/rapm-2022-103907 35998937

[20] Reysner T, Kowalski G, Reysner M, Lapaj L, Daroszewski P, Wieczorowska-Tobis K. Erector spinae plane block (ESPB) vs. pericapsular nerve group (PENG) block in total hip arthroplasty in elderly patients: a randomized, double-blinded, controlled trial. Anaesthesiol Intensive Ther. 2025;57(1):90–8. 10.5114/ait/203170. 40420611 PMC12210365

[21] Janssens WH, Verhoestraete P, Piers RD, Van Den Noortgate NJ. Short-term opioid treatment of acute locomotor pain in older adults: comparison of effectiveness and safety between tramadol and oxycodone: a randomized trial. Geriatrics. 2024;9(2):46. 38667513 10.3390/geriatrics9020046 PMC11050500

[22] Bilek AJ, Cullen S, Carolyn MT, Qixuan L, Huszti E, Norman RE. Opioid use in older orthopedic rehabilitation inpatients: a retrospective study. Eur J Phys Rehabil Med. 2023;59(2):192. 36745157 10.23736/S1973-9087.23.07650-5 PMC10170320

[23] Rathee A, Chaurasia MK, Singh MK, Singh V, Kaushal D. Relationship between pre-and post-operative C-Reactive Protein (CRP), Neutrophil-to-Lymphocyte Ratio (NLR), and Platelet-to-Lymphocyte Ratio (PLR) with post-operative pain after total hip and knee arthroplasty: an observational study. Cureus. 2023;15(8):e43782. 37731439 10.7759/cureus.43782 PMC10507425

[24] Seifert O, Baerwald C. Interaction of pain and chronic inflammation. Z Rheumatol. 2021;80(3):205–13. 33373022 10.1007/s00393-020-00951-8

[25] Kukreja P, Uppal V, Kofskey AM, Feinstein J, Northern T, Davis C, et al. Quality of recovery after pericapsular nerve group (PENG) block for primary total hip arthroplasty under spinal anaesthesia: a randomised controlled observer-blinded trial. Br J Anaesth. 2023;130(6):773–9. 36964012 10.1016/j.bja.2023.02.017

[26] Zotova N, Zhuravleva Y, Chereshnev V, Gusev E. Acute and chronic systemic inflammation: features and differences in the pathogenesis, and integral criteria for verification and differentiation. Int J Mol Sci. 2023;24(2):1144. 36674657 10.3390/ijms24021144 PMC9862412

[27] Powell JE, Boehm JO, Bicher JH, Reece CL, Davis SA, Pasquina PF. The utility of dynamic movement orthoses in the management of complex regional pain syndrome—A case series. Mil Med. 2023;188(7–8):e2712–8. 10.1093/milmed/usab418 34626479

[28] Rahimi Mehdi Abad F, Khalili P, Jalali F, Pirsadeghi A, Esmaeili Nadimi A, Manshoori A, et al. Maternal opioid use is reflected on leukocyte telomere length of male newborns. PLoS ONE. 2021;16(12):e0261013. 34919564 10.1371/journal.pone.0261013 PMC8682876

[29] Chen A, Kolodzie K, Schultz A, Hansen EN, Braehler M. Continuous lumbar plexus block vs continuous lumbar erector spinae plane block for postoperative pain control after revision total hip arthroplasty. Arthroplast Today. 2021;9:29–34. 33997205 10.1016/j.artd.2021.03.016 PMC8099915

[30] Indra B, Lipoeto NI, Tjong DH, Rahman S. Alteration of interleukin-4, interleukin-6 levels, and post-operative pain intensity. Open Access Maced J Med Sci. 2023;11(A):1–7.

